# Rare variants of DNA ligase 1 show distinct mechanisms of deficiency

**DOI:** 10.1016/j.jbc.2024.107957

**Published:** 2024-11-05

**Authors:** Jenna H. Veenstra, Alexandria Chabez, Terrance J. Haanen, Austin Keranen, Charlotte Cunningham-Rundles, Patrick J. O’Brien

**Affiliations:** 1 Department of Biological Chemistry, University of Michigan, Ann Arbor, Michigan, USA; 2 Department of Medicine, Division of Clinical Immunology, Icahn School of Medicine at Mount Sinai, New York, New York, USA

**Keywords:** LIG1 syndrome, IMD96, DNA ligase, immunodeficiency, DNA ligation

## Abstract

Human DNA ligase 1 (LIG1) performs the final step in DNA repair and recombination pathways by sealing DNA breaks, and it functions as the main replicative ligase. Hypomorphic LIG1 variants R771W and R641L cause immune deficiencies in LIG1 Syndrome patients. *In vitro* these LIG1 variants have decreased catalytic efficiency and increased abortive ligation and it is not known if either biochemical defect is sufficient on its own to cause immune deficiency. We investigated the enzymatic activity of several new candidate LIG1 Syndrome variants chosen based on their structural proximity to known clinical variants, low minor allele frequency (MAF), high level of conservation, and concurrence in patients with similar symptoms as LIG1 Syndrome patients. The R305Q substitution is in the DNA binding domain, R768W is in the OB-fold domain, and R641S is in the nucleotidyltransferase domain. Biochemical characterization confirmed deficiencies in ligase activity for all three variants, but also revealed marked differences in comparison to the known LIG1 Syndrome variants. Both the R305Q and R768W substitutions increase the K_M_ for DNA and decrease the catalytic efficiency, however, neither exhibit elevated levels of abortive ligation. In contrast, the R641S variant exhibits a greater impairment of activity as well as a more pronounced level of abortive ligation compared to the known LIG1 Syndrome variant, R641L. This work expands the number of LIG1 alleles that are likely candidates for LIG1 Syndrome, and it raises the question of whether distinct enzymatic deficiencies in LIG1 cause unique clinical impacts in patients harboring these alleles.

Human DNA ligase 1 (LIG1) is responsible for completing nuclear DNA replication and DNA repair pathways including nucleotide excision repair, mismatch DNA repair, and long-patch base excision repair. LIG1 is essential in mammals (1), but cells lacking LIG1 remain viable as other DNA ligases can compensate for the loss of LIG1 (2, 3, 4). A single copy of LIG1 is sufficient for normal development, because several individuals with a single functional allele have been described (5). However, individuals with biallelic hypomorphic LIG1 alleles suffer from symptoms of immune deficiency (5, 6, 7).

LIG1 has three highly conserved domains that constitute the catalytic core of the enzyme (Fig. 1*A*); however, the N-terminal region (1–160) is predicted to be unstructured (8, 9). This region is necessary for cellular function as it contains a nuclear localization sequence and a motif for interaction with PCNA (9, 10, 11). *In vitro*, the N-terminal unstructured region is dispensable for ligase activity. The DNA-binding domain (DBD) increases the affinity for DNA and is required for efficient DNA ligation (8). The nucleotidyltransferase (NTase) domain contains many of the active site residues required for chemistry, including K568 which harbors the covalent AMP-lysyl intermediate. The oligosaccharide binding domain (OBD) makes contact with the DNA substrate but also has conserved catalytic residues required for the adenylation of the NTase domain. These three domains encircle the DNA substrate, and there are extensive contacts between the DBD and the OBD as well as between the DBD and the NTase domain and between the NTase domain and the OBD (8).

**Figure 1:**
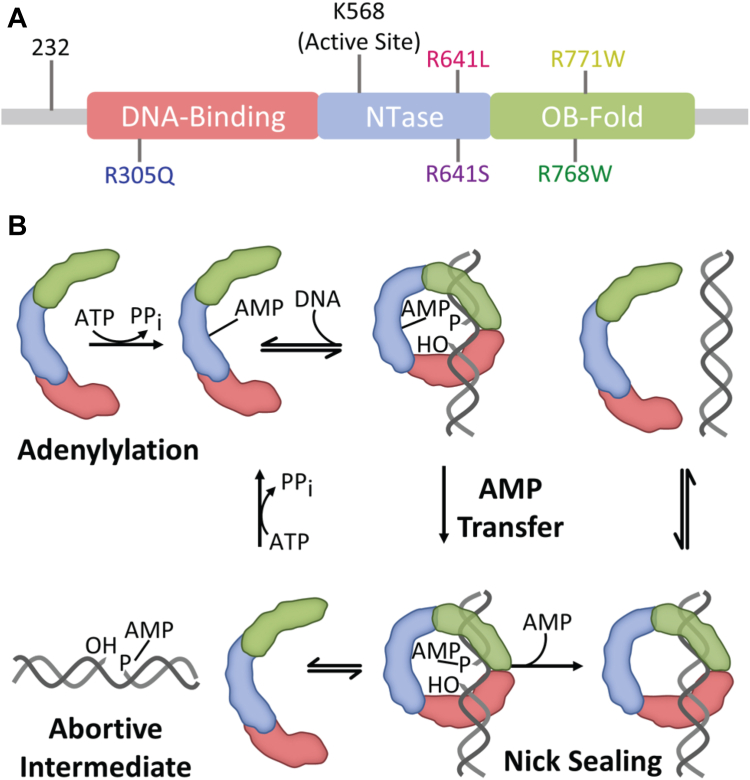
**DNA Ligase I domain architecture and mechanism.***A*, the structure of LIG1 consists of an N-terminal disordered region, and three catalytic domains: the DNA-binding domain (DBD, *red*), the nucleotidyltransferase (NTase) domain (*blue*), and the oligonucleotide binding domain (OBD, *green*). The previously characterized LIG1 Syndrome variants are listed above the schematic and the new putative LIG1 Syndrome mutations are indicated below. *B*, the mechanism of LIG1-catalyzed DNA ligation starts with the adenylation of LIG1 at the active site lysine residue. The AMP group is then transferred to the 5′-phosphate of a nicked DNA substrate. The 3′-hydroxyl of the nick attacks the 5′-phosphate, sealing the nick and releasing AMP. During abortive ligation, LIG1 falls off the AMP-DNA intermediate and is adenylated again, precluding rebinding.

The three-step reaction mechanism of DNA ligases to ligate single-strand breaks is conserved across evolution (Fig. 1*B*) (12, 13). LIG1 is first adenylylated by ATP at K568 in the active site to release inorganic pyrophosphate. Second, the 5′-phosphate at the nick attacks the lysyl-AMP to create a 5′-5′ phosphoanhydride bond. Third, the 3′-hydroxyl attacks the 5′-phosphate of the DNA creating the new phosphodiester bond and releasing the AMP group. LIG1 may prematurely release the AMP-DNA intermediate in a process known as abortive ligation (14, 15). LIG1 is rapidly re-adenylylated under physiological conditions, and therefore, the AMP group must be removed from the abortive intermediate either by the direct action of the hydrolase aprataxin or *via* strand displacement synthesis. LIG1 requires Mg^2+^ to perform each enzymatic step, and at a decreased concentration of Mg^2+^, the WT enzyme exhibits increased abortive ligation (15). One Mg^2+^ ion is bound within the active site where it functions as a catalytic metal ion. Its placement allows for the stabilization of the pentavalent transition state that occurs during the nick-sealing reaction (16). This catalytic Mg^2+^ binding site is conserved amongst all DNA ligases (17, 18). A second Mg^2+^ ion is coordinated between the DBD, the NTase, and the backbone of the DNA. This site is unique to LIG1 and has been dubbed the HiFi Mg^2+^ ion because it confers high fidelity (16). The HiFi Mg^2+^ ion enforces the structural rearrangement of the 3′-strand such that 3′-mismatches can be rejected prior to ligation as abortive ligation intermediates.

Rare disease-causing variants are found in the *LIG1* gene. LIG1 Syndrome (also known as IMD96; Immunodeficiency 96) was characterized in the early 1990s as a genetic deficiency in LIG1 (6, 7). The original patient exhibited developmental defects, UV sensitivity, and immunodeficiency. Subsequently, five more patients were identified with rare variants in the *LIG1* gene, and these individuals also showed symptoms of immunodeficiency (5). The causative mutations were biallelic, and in some cases one copy is inactive and the other is biochemically impaired. The catalytically impaired variants are R771W and R641L which are on DNA-binding loops in the OB-fold and NTase domains, respectively (Fig. 1*A*). Both substitutions decrease the catalytic efficiency and increase abortive ligation when compared to the WT enzyme, and both defects are partially rescued at very high concentrations of Mg^2+^ (5, 19). Although both residues are located on DNA binding loops, there is not a large defect in the overall DNA binding affinity of either LIG1 variant (19). As these previously characterized mutations lead to both increased abortive ligation and decreased catalytic efficiency, it is not known whether the unique pathologies of LIG1 Syndrome are caused by the higher load of abortive ligation or the decreased kinetics of ligation in replication and repair pathways. It is expected that additional symptomatic, rare *LIG1* variants exist, but to date, there is little publicly available information.

To address these unknowns, we identified several rare *LIG1* variants as candidates for LIG1 Syndrome (Fig. 1*A*). First, a patient exhibiting similar symptoms as those with LIG1 Syndrome was reported to be homozygous for R305Q LIG1 (20). This patient’s symptoms are consistent with the clinical description of LIG1 deficiency which includes immunodeficiency, dysmorphic features, and growth delay. The R305 residue is highly conserved in the DBD and makes a network of interactions in the minor groove of the DNA. Although it was originally reported that the R305Q variant results in complete loss of LIG1 protein (20), this seemed unlikely because deletion of *LIG1* in mice is embryonic lethal (4). Rather, we considered the hypothesis that this substitution may disrupt DNA binding in a mechanism distinct from the previously characterized variants. Additionally, using an exome sequence database of patients with primary immune deficiency, we found several rare mutations in *LIG1* and selected R641S and R768W for investigation as they are proximal to the previously characterized mutations.

We purified these candidate LIG1 variants and performed biochemical analyses to determine their relative DNA binding affinity and catalytic efficiency. Under a physiologically relevant condition of 1 mM free Mg^2+^, we found that the R305Q candidate variant causes a 25-fold decrease in catalytic efficiency, which is similar to the defect observed previously for the R771W variant. However, we did not detect elevated abortive ligation for the R305Q LIG1 variant, and this mutation disrupts DNA substrate binding, making it distinct from the known LIG1 Syndrome variants. We found that R641S exhibited increased abortive ligation and an order of magnitude greater loss in catalytic efficiency when compared to R641L. The R768W variant was surprising in that it showed a similar defect in catalytic efficiency as R771W; however, it lacks the abortive ligation seen for R771W. This study shows that decreased efficiency, reduced substrate affinity, and elevated abortive ligation are not necessarily coupled for LIG1 clinical variants. The unique biochemical properties of the LIG1 variants studied raise the possibility that these alleles confer distinct cellular consequences.

## Results

### Active site titration and stability of LIG1 mutant proteins

**Figure 2:**
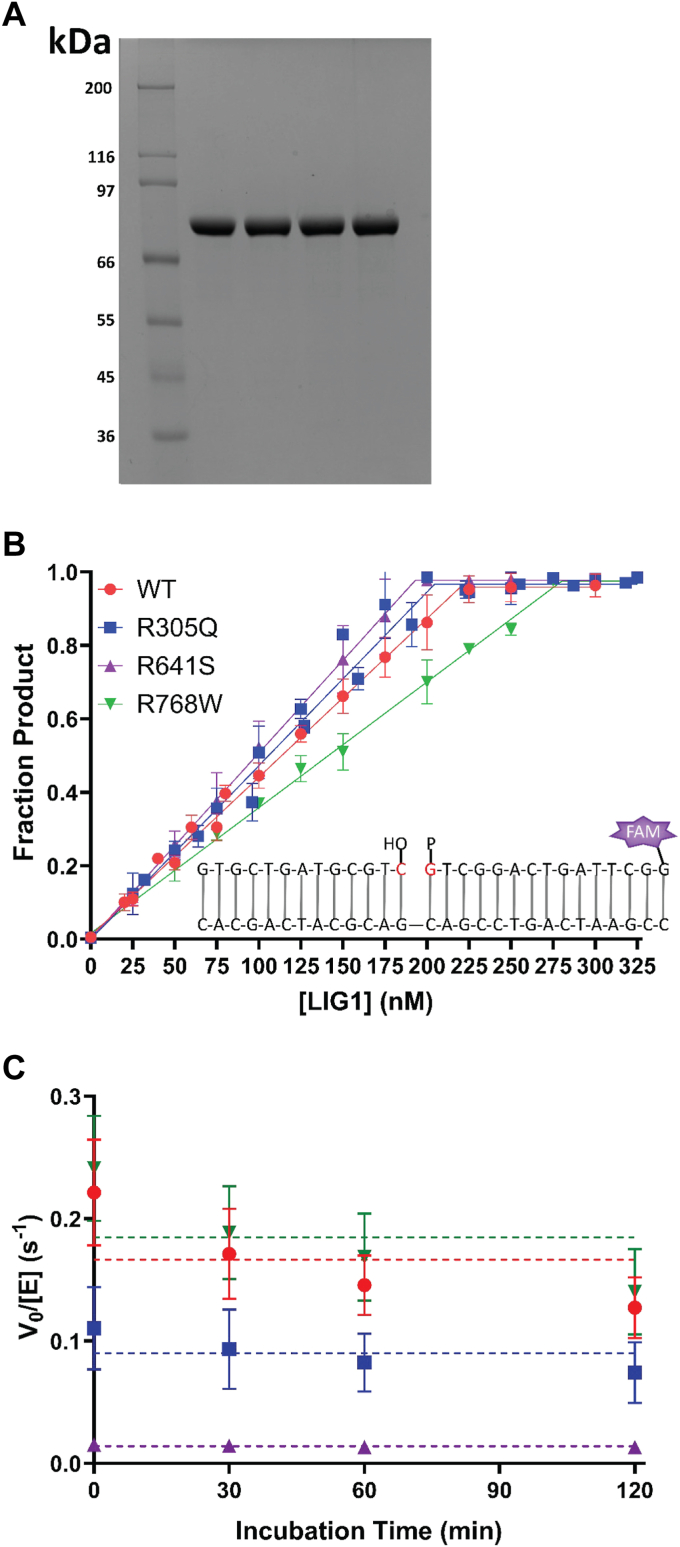
**Determination of active concentration and stability of Δ232 LIG1 variants.***A*, purified Δ232 LIG1 proteins (1 mg per lane) were analyzed on a 10% SDS-PAGE gel. Lane one contains MW standards. Lanes 2 to four are WT, R305Q, R641S, and R768W, respectively. *B*, active site titrations were performed for each variant and WT LIG1 at the indicated amount with 150 nM nicked DNA substrate (inset) in the absence of ATP. The fits give active concentrations of 68%, 73%, 78%, and 53%, respectively. The inset is a representation of the 28-mer nicked DNA substrate used. *C*, the stability of LIG1 variants was determined by incubating the protein for the indicated amount of time before adding substrate and measuring steady-state ligase activity. All variants were stable for the duration of the incubation. All values are mean ± SD (N ≥ 3).

LIG1 has an extensive unstructured amino-terminus and therefore we used an N-terminal truncation (Δ232) that retains full catalytic activity and has been previously characterized (8, 15). Wild-type (WT) and variants of LIG1 were expressed in *Escherichia coli* and purified as previously described (5). Each of the variants was expressed in similar yields as the WT enzyme and was estimated to have a purity of >95% (Fig. 2*A*). To quantitatively compare novel LIG1 variants to the WT LIG1, it is critical to accurately determine the active concentration of the enzyme. LIG1 is purified as the adenylylated protein which allows for the use of single-turnover ligation in the absence of ATP to measure the amount of active enzyme (15). Active site titration reactions were performed with a fixed concentration of 150 nM nicked DNA substrate to obtain the active concentration of each protein (Fig. 2*B*). The percentage of active enzymes varied in the range of 53% to 78%. Subsequently, we corrected for these small differences between preparations, and the indicated concentrations throughout reflect those determined by active site titration.

Previous work established that LIG1 requires ≥5 mM concentration of free Mg^2+^ ions for maximal stimulation of steady-state DNA ligation activity (15). Although total intracellular pools of cellular Mg^2+^ ions may exceed 30 mM, most of this pool is bound with micromolar affinity and the freely available concentration of Mg^2+^ has been measured to be between 0.3 and 1.5 mM for both microbial and eukaryotic cell types (21, 22). As with previous work, we chose to employ 1 mM free Mg^2+^ as a reasonable approximation of physiologically relevant concentration of Mg^2+^ (5, 15, 19).

Ligation reactions were performed with 0.2 mM ATP, 1 mM free Mg^2+^, at pH 7.5, and with an ionic strength of 150 mM. To evaluate the stability of each enzyme under these reaction conditions, LIG1 was incubated at 37 °C without the DNA substrate for up to 2 hours, at which point, the reaction was initiated by introducing the DNA substrate, and the initial rates of ligation were measured. Although each variant showed a different amount of ligase activity, this activity was retained for up to 2 hours, indicating that all mutants were sufficiently stable for kinetic analysis which is completed in less than 2 hours (Fig. 2*C*).

### Substrate dependence of LIG1 variants

**Figure 3:**
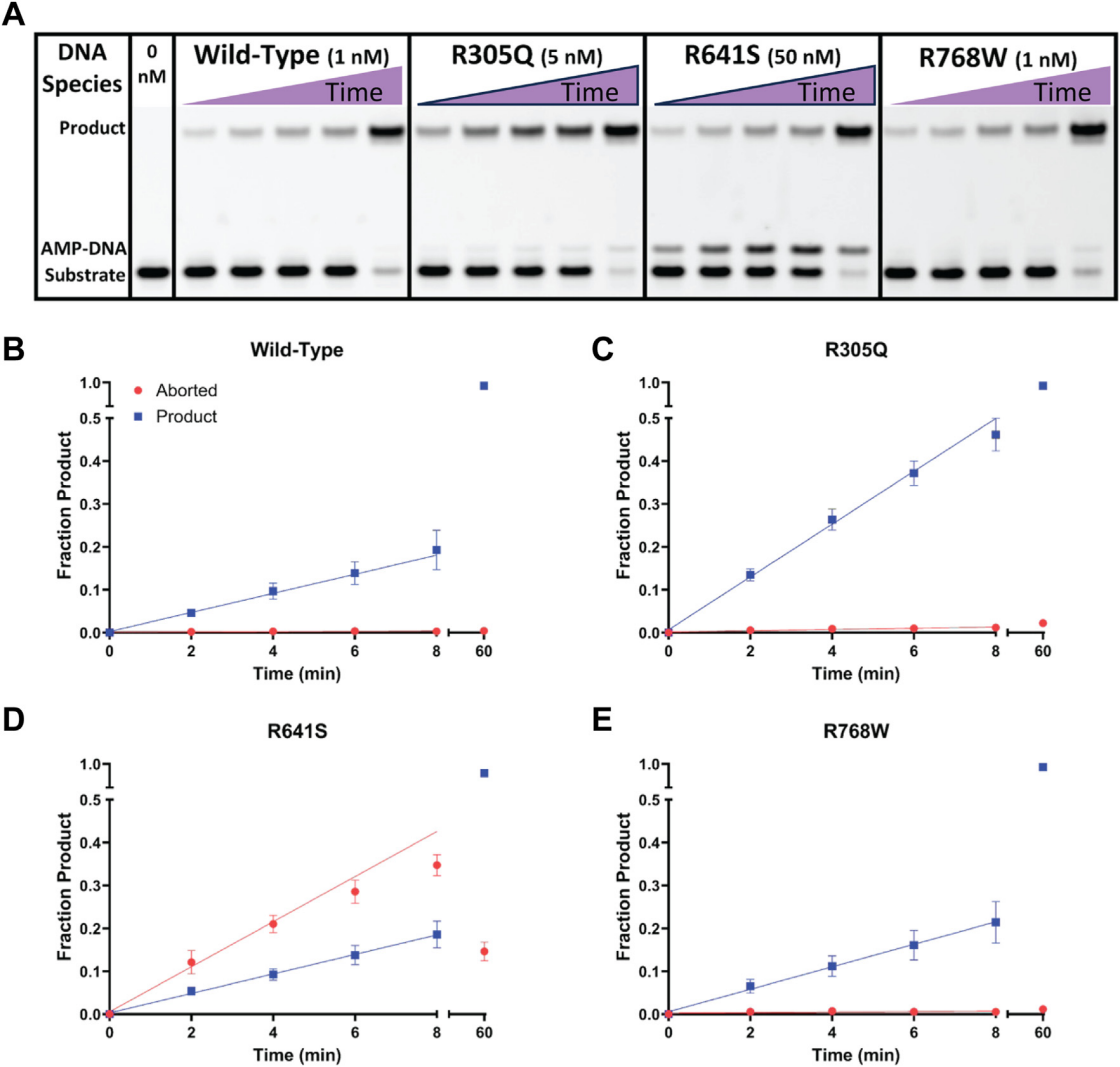
**Representative initial rates for ligation by LIG1 variants.***A*, representative gel for the indicated concentration of each LIG1 variant. The time course for the corresponding portion of the gel for WT (*B*), R305Q (*C*), R641S (*D*), and R768W (*E*). *A−E*, reactions included 1000 nM nicked DNA substrate, 0.2 mM ATP and 1 mM free Mg^2+^. The first lane on the gel is a no enzyme control. The linear regressions for the ligated product (*blue*) and aborted AMP-DNA (*red*) are determined from the linear portion of the time courses (the final points are excluded from the fits). All values are mean ± SD (N = 3).

To estimate the differences in ligase activity for each variant, steady-state ligation assays were conducted with limiting enzyme and excess (1 μM) 28mer nicked DNA substrate. These results are summarized in Figure 3 which includes a representative gel showing the product, abortive intermediate, and substrate bands from top to bottom. The initial velocities were calculated from the plots of the fraction of product *versus* time (Fig. 3, *B–D*). The range of LIG1 concentrations used for this assay was 50-fold (1–50 nM) to account for the large differences in ligation activity between the WT and mutant enzymes. From these preliminary experiments, it was clear that all variants were active DNA ligases; however, the maximal rate of ligation varied between them and the R641S variant showed a large amount of abortive ligation (Fig. 3*A*).

We next used steady-state kinetics to investigate the ATP dependence of the LIG1 variants, varying the ATP concentration from 0.25 to 200 μM at saturating conditions of free Mg^2+^ (20 mM) (Fig. S1 and Table 1). The use of saturating concentrations of Mg^2+^ avoids complications from ATP sequestering the free Mg^2+^ that is needed for catalysis (15). These experiments revealed that each mutant had only modest changes to k_cat_/K_M,ATP_ compared to the WT enzyme, with R768W having the largest defect of approximately two-fold. The known LIG1 Syndrome variants also showed little to no changes to the value of k_cat_/K_M,ATP_ relative to WT LIG1 (Table 1) (19). These data indicate that the ATP-dependent step is not significantly altered by the LIG1 Syndrome variants that predominantly impact the DNA-dependent steps of the reaction.

**Table 1:** Steady-state ATP dependence for LIG1 variants^a^

Rate constant	WT	R305Q	R641S	R768W	R771W	R641L
k_cat_ (s^−1^)	0.64 ± 0.01	0.44 ± 0.02	0.07 ± 0.002	0.37 ± 0.01	0.19 ± 0.02	0.27 ± 0.02
K_M,ATP_ (μM)	14 ± 1.2	8.4 ± 1.9	1.7 ± 0.3	15 ± 1.6	4.0 ± 0.3	5.8 ± 0.3
k_cat_/K_M,ATP_ (M^−1^s^−1^)	4.6 ± 0.4 × 10^4^	5.2 ± 1.2 × 10^4^	4.3 ± 0.7 × 10^4^	2.5 ± 0.3 × 10^4^	4.8 ± 0.3 × 10^4^	4.6 ± 0.3 × 10^4^
Relative k_cat_/K_M,ATP_	(1)	1.1	0.94	0.55	1.0	1.0

a Steady-state kinetics were determined at 37 °C using saturating Mg^2+^_free_ (20 mM), 1 to 10 nM LIG1, and 1000 nM DNA substrate (Fig. S1). Values for R641L and R771W were taken from Jurkiw *et al.* (19). All values are the mean ± SD (N ≥ 3).

**Figure 4:**
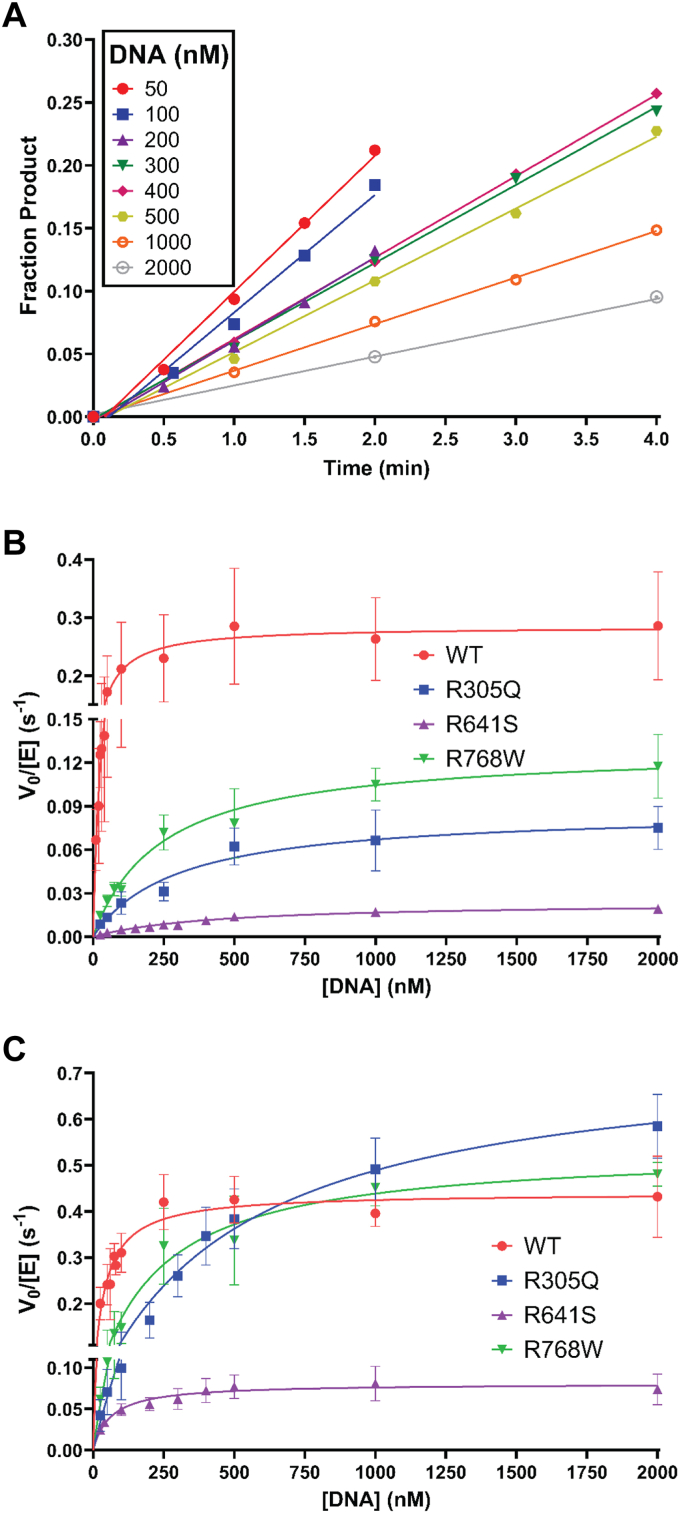
**Steady-state DNA dependence of LIG1 variants.***A*, representative time courses from steady-state reactions containing 43 nM R641S, 1.2 mM MgCl_2_ (1 mM Mg^2+^_free_), 0.2 mM ATP, and the indicated DNA substrate concentrations. *B* and *C*, the DNA dependence was determined with 0.4 to 43 nM LIG1. These data were fit by the Michaelis-Menten equation to obtain k_cat_*and* K_M,DNA_ values for WT and variant LIG1 enzymes. DNA dependence was performed at (*B*) physiological conditions of 1 mM Mg^2+^_free_ and 0.2 mM ATP or (*C*) saturating conditions of 20 mM Mg^2+^_free_ and 1 mM ATP. Values are the mean ± SD (N ≥ 3). Kinetic parameters are summarized in Table 2.

We then measured the steady-state nicked DNA substrate dependence for each variant and compared them to the parameters for the WT enzyme. We started with physiological levels of free Mg^2+^ (1 mM). Representative time courses for initial rate determination can be found in Figure 4*A* and the Michaelis-Menten substrate dependence for each variant is shown in Figure 4*B*. The catalytic efficiency for the DNA substrate (k_cat_/K_M_) of each variant is summarized in Table 2. The R305Q variant shows a 26-fold defect in catalytic efficiency, that is similar to the previously determined defect of R641L (20-fold) and R771W (33-fold), the known LIG1 Syndrome variants (19). The R641S variant has a greater effect on catalytic efficiency than R641L with a respective ∼125-fold decrease *versus* the 20-fold decrease when compared to WT LIG1. The R768W variant shows the smallest defect of the tested mutants, with a decrease in catalytic efficiency of 14-fold, similar to the 20-fold defect in the previously studied R641L variant (19). The differences in k_cat_/K_M_ are due to changes in both K_M, DNA_ and k_cat_ (Fig. 4*B* and Table 2). The R305Q and R768W substitutions decreased the maximal turnover number (k_cat_) by about half whereas the R641S substitution decreased the k_cat_ value by more than 10-fold. The K_M, DNA_ values for the LIG1 variants are between 4- and 10-fold higher than that of the WT enzyme (Table 2). These data support the initial hypothesis that each of the new variants is catalytically compromised for DNA ligation; however, the enzymatic impact differs for each variant.

**Table 2:** Steady-state DNA substrate dependence for LIG1 variants^a^

Rate constant	WT	R305Q	R641S	R768W	R771W	R641L
1 mM Mg^2+^						
k_cat_ (s^−1^)	0.28 ± 0.02	0.09 ± 0.006	0.024 ± 0.0001	0.13 ± 0.007	0.041 ± 0.002	0.066 ± 0.009
K_M_ (nM)	37 ± 8.6	299 ± 72	433 ± 43	241 ± 40	220 ± 74	211 ± 67
k_cat_/K_M_ (M^−1^s^−1^)	7.7 ± 1.9 × 10^6^	2.9 ± 0.7 × 10^5^	5.5 ± 0.6 × 10^4^	5.4 ± 0.9 × 10^5^	2.2 ± 0.8 × 10^5^	3.6 ± 1.6 × 10^5^
Relative k_cat_/K_M_	(1)	0.04	0.007	0.07	0.03	0.05
20 mM Mg^2+^						
k_cat_ (s^−1^)	0.44 ± 0.02	0.75 ± 0.03	0.08 ± 0.003	0.53 ± 0.02	0.20 ± 0.03	0.31 ± 0.02
K_M_ (nM)	37 ± 6	537 ± 60	62 ± 11	219 ± 32	128 ± 10	82 ± 14
k_cat_/K_M_ (M^−1^s^−1^)	1.2 ± 0.2 × 10^7^	1.4 ± 0.2 × 10^6^	1.3 ± 0.2 × 10^6^	2.4 ± 0.4 × 10^6^	1.6 ± 0.3 × 10^6^	3.8 ± 0.7 × 10^6^
Relative k_cat_/K_M_	(1)	0.12	0.11	0.21	0.13	0.32

a Steady-state kinetics were determined at 37 °C using 0.4 to 43 nM LIG1 at either physiological (1 mM Mg^2+^_free_ and 0.2 mM ATP) concentrations or saturating (20 mM Mg^2+^_free_ and 1 mM ATP) concentrations (Fig. 4). Values for R641L and R771W taken from Maffucci *et al.* and Jurkiw *et al.* (5, 19). Values are the mean ± SD (N ≥ 3).

Previously, it was found that the known LIG1 Syndrome variants could be partially rescued by increasing the concentration of free Mg^2+^ to saturating levels (20 mM) (19). Therefore, we determined the DNA dependence again at this higher concentration of Mg^2+^ to evaluate if these novel variants behaved similarly (Fig. 4*C* and Table 2). While there was partial rescue of steady-state ligation activity across the board, the extent of rescue differed from that seen with the previously described R641L and R771W variants. Notably, both R305Q and R768W were completely rescued with regards to the k_cat_ values, but neither of their K_M, DNA_ values were rescued. Indeed, the R305Q variant exhibited a 2-fold higher K_M, DNA_ value at saturating Mg^2+^ concentrations, as compared to the 1 mM Mg^2+^ condition. Nevertheless, due in part to k_cat_ values that exceed that of the WT enzyme, the R305Q and R768W variants were partially rescued to within 5 – 8-fold that of the WT enzyme which is in the same range as observed for the R641L and R771W variants at the high Mg^2+^ condition. The R641S variant showed rescue of both k_cat_ and K_M, DNA_ such that at high Mg^2+^ the defect in k_cat_/K_M_ is in line with the defects of the other variants (Table 2). In summary, these new LIG1 variants were rescued by increasing the concentration of Mg^2+^; however, the manner and extent of rescue differed to some extent from the known LIG1 Syndrome variants.

### Mg^2+^ dependence and abortive ligation

**Figure 5:**
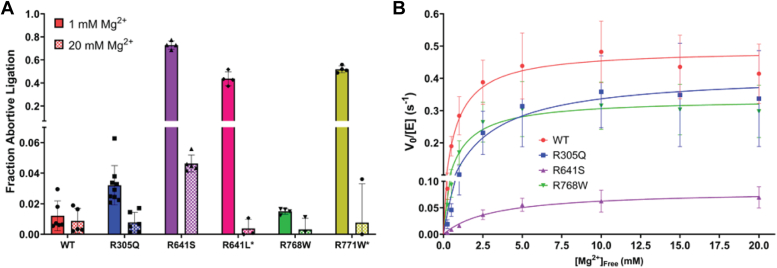
**Abortive ligation and steady-state Mg**^**2+**^**dependence.***A*, fraction abortive ligation was calculated at physiological (1 mM) or saturating (20 mM) concentrations of Mg^2+^_free_. Values for R641L and R771W were taken from Maffucci *et al.* and Jurkiw *et al.* (5, 15). *B*, steady-state Mg^2+^ dependence was determined with 1 to 43 nM LIG1, 1000 nM DNA substrate, 0.2 mM ATP. These data were fit by the Michaelis-Menten equation to obtain *k*_*cat*_ and *K*_*Mg*_ values for WT and variant LIG1 enzymes. Values are the mean ± SD (N ≥ 3) and kinetic constants are presented in Table 3.

A striking characteristic of the known LIG1 Syndrome variants is the elevated abortive ligation that they perform (5). By comparing the magnitude of abortive ligation at physiological *versus* saturating levels of free Mg^2+^, we found that these new variants do not all act like the known variants (Fig. 5*A*). Namely, the R768W variant has very little abortive ligation at both 1 and 20 mM Mg^2+^ concentrations. The R305Q variant has slightly elevated abortive ligation levels compared to WT LIG1 at physiological Mg^2+^ concentration, but not to the same level as the known LIG1 Syndrome variants (Fig. 5*A*). R641S behaves similarly to the known LIG1 Syndrome variant, R641L, with even more abortive ligation at physiological Mg^2+^ concentration. R641L LIG1 retains elevated abortive ligation even at saturating Mg^2+^ concentration.

To further evaluate the impact the LIG1 mutations have on the utilization of the Mg^2+^ cofactor, we determined the Mg^2+^ dependence of steady-state ligation at fixed, saturating concentrations of both ATP and DNA substrates (Fig. 5*B*). These data were fit by the equation for a single Mg^2+^ binding event to yield the apparent binding constant for Mg^2+^ (K_Mg_) and the overall efficiency of Mg^2+^ utilization (k_cat_/K_Mg_), and the values are summarized in Table 3. Of the new variants, R641S showed the largest increase in K_Mg_ of almost 4-fold and an overall decrease in k_cat_/K_Mg_ of 25-fold as compared to WT LIG1 (Table 3). The other two variants, R305Q and R768W, which do not show increased abortive ligation, showed more modest defects in the utilization of Mg^2+^ with R305Q and R768W showing only 2– 4-fold decreases in k_cat_/K_Mg_ (Table 3). However, the R768W variant is the only one of the variants studied to not show a significantly elevated K_Mg_ value (the measured value of 1.0 ± 0.2 mM for R768W is within error of the 0.77 ± 0.15 mM value for WT LIG1; Table 3). These data indicate that most, but not all, of the hypomorphic alleles of LIG1, are impacted by a deficiency in Mg^2+^ binding that compromises ligation activity in the physiologically relevant Mg^2+^ concentration range.

**Table 3:** Steady-state magnesium ion dependence for LIG1 variants^a^

Rate constant	WT	R305Q	R641S	R768 W	R771W	R641L
k_cat_ (s^−1^)	0.49 ± 0.02	0.41 ± 0.03	0.08 ± 0.001	0.34 ± 0.02	0.18 ± 0.03	0.27 ± 0.05
K_Mg_ (mM)	0.77 ± 0.15	2.3 ± 0.6	3.2 ± 1.0	1.0 ± 0.2	3.5 ± 0.4	4.3 ± 1.5
k_cat_/K_Mg_ (M^−1^s^−1^)	630 ± 130	180 ± 50	26 ± 9	340 ± 80	51 ± 10	63 ± 12
Relative k_cat_/K_Mg_	(1)	0.28	0.04	0.54	0.08	0.10

a Steady-state kinetics were determined at 37 °C using saturating ATP (0.2 mM), 1 to 43 nM LIG1, and 1000 nM DNA substrate (Fig. 5). Values for R641L and R771W are from Jurkiw *et al.* (19). Values are the mean ± SD (N ≥ 3).

### Equilibrium DNA binding

**Figure 6:**
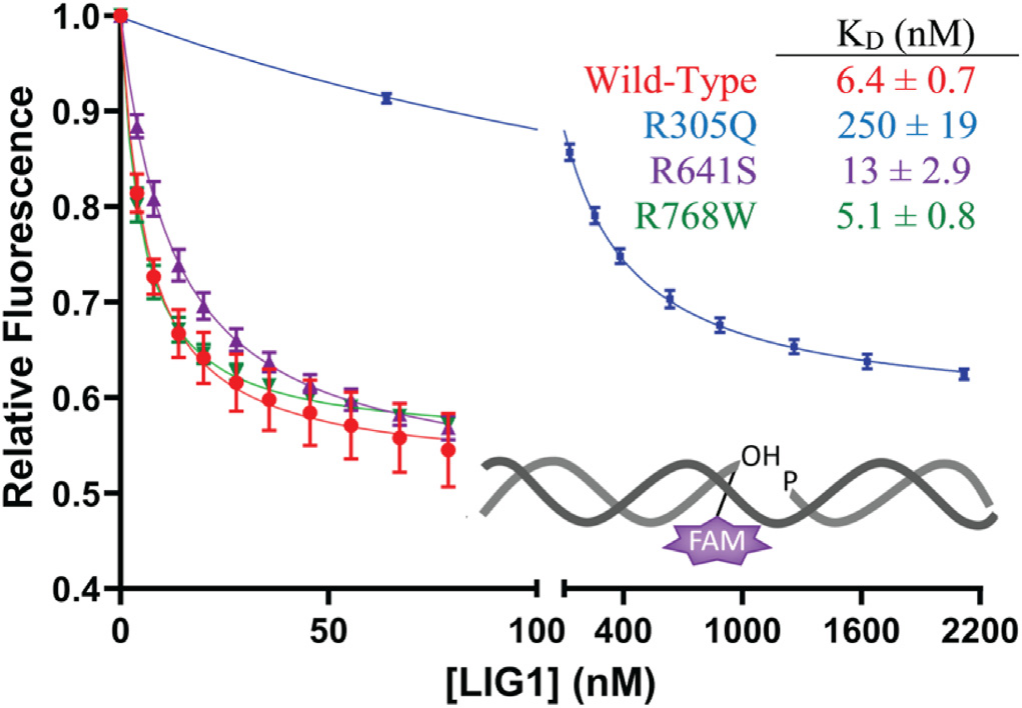
**Equilibrium binding of LIG1 variants to a nicked DNA substrate.** The binding of the LIG1 enzymes to the DNA was monitored using fluorescence quenching in the absence of Mg^2+^ to prevent ligation. The inset shows the oligonucleotide used to measure DNA binding contains a fluorescein label conjugated to a deoxythymidine base (TFAM) at the 3′ position of the nick site. Data were fit by a simple binding hyperbola and the *K*_*D*_ values are provided in the inset (mean ± SD, N ≥ 3).

To directly interrogate potential defects in DNA binding by these LIG1 variants, we used a reporter DNA substrate containing fluorescein-conjugated deoxythymidine at the nick (TFAM; Fig. 6). We monitored the quenching of fluorescence at fixed DNA concentration and increasing concentration of LIG1 in the absence of Mg^2+^ to prevent DNA ligation and to measure equilibrium binding to nicked DNA (Fig. 6). For WT LIG1, we measured a K_D_ value of 6.4 ± 0.7 nM. R768W LIG1 does not have a defect in DNA binding with a K_D_ value of 5.7 ± 0.4 nM. R641S LIG1 has a modest defect in DNA binding with a K_D_ value approximately 2-fold higher than that of the WT enzyme. This defect is very similar to the 1.4 – 1.6-fold weaker DNA binding measured previously for R771W and R641L (19). Interestingly, R305Q LIG1 has a very large defect with a K_D_ value of 250 ± 19, which is an almost 40-fold increase compared to WT LIG1. This strong impact of the R305Q substitution on binding to nicked DNA distinguishes it from all the other LIG1 syndrome-associated variants (Fig. 6).

## Discussion

**Figure 7:**
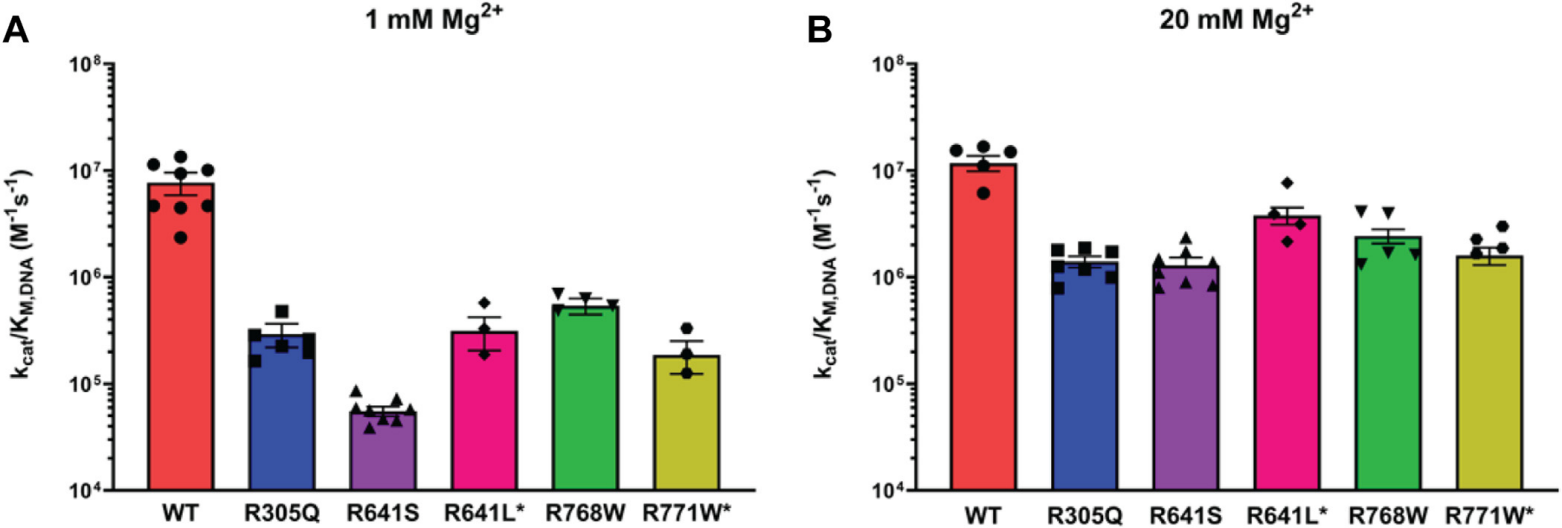
**Summary of the catalytic efficiency for LIG1 variants.** Catalytic efficiency (*k*_*cat*_*/K*_*M*_) values for 1 mM (*A*) and 20 mM Mg^2+^_free_ (*B*) were calculated from the data in Table 2. Values are the mean ± SD (N ≥ 3).

Previous work on LIG1 Syndrome showed that the R641L and R771W LIG1 variants cause a substantial decrease in catalytic efficiency (19). Both variants also show weakened Mg^2+^ affinity and an increase in abortive ligation, but they do not exhibit a strong defect in DNA substrate binding. It was proposed that the abortive ligation caused by R641L and R771W may contribute to the physiological symptoms because alleles with abortive ligation were common to all known patients at the time (19). In this report, we determined kinetic parameters for additional candidate LIG1 Syndrome variants enabling us to test the previously observed correlation between abortive ligation, reduced Mg^2+^ affinity, and decreased catalytic efficiency. These three new candidate LIG1 alleles all have compromised biochemical activities, however, we observe a wider range of biochemical defects and, most notably, found that the link between abortive ligation and reduced catalytic activity is broken in the R768W variant which is adjacent to the previously described R771W variant and in the R305Q variant which is the first candidate disease allele located within the DNA binding domain. The changes in catalytic efficiency for the known and candidate LIG1 Syndrome variants are summarized in Figure 7 and will be discussed below.

### R641S is more severe than R641L

**Figure 8:**
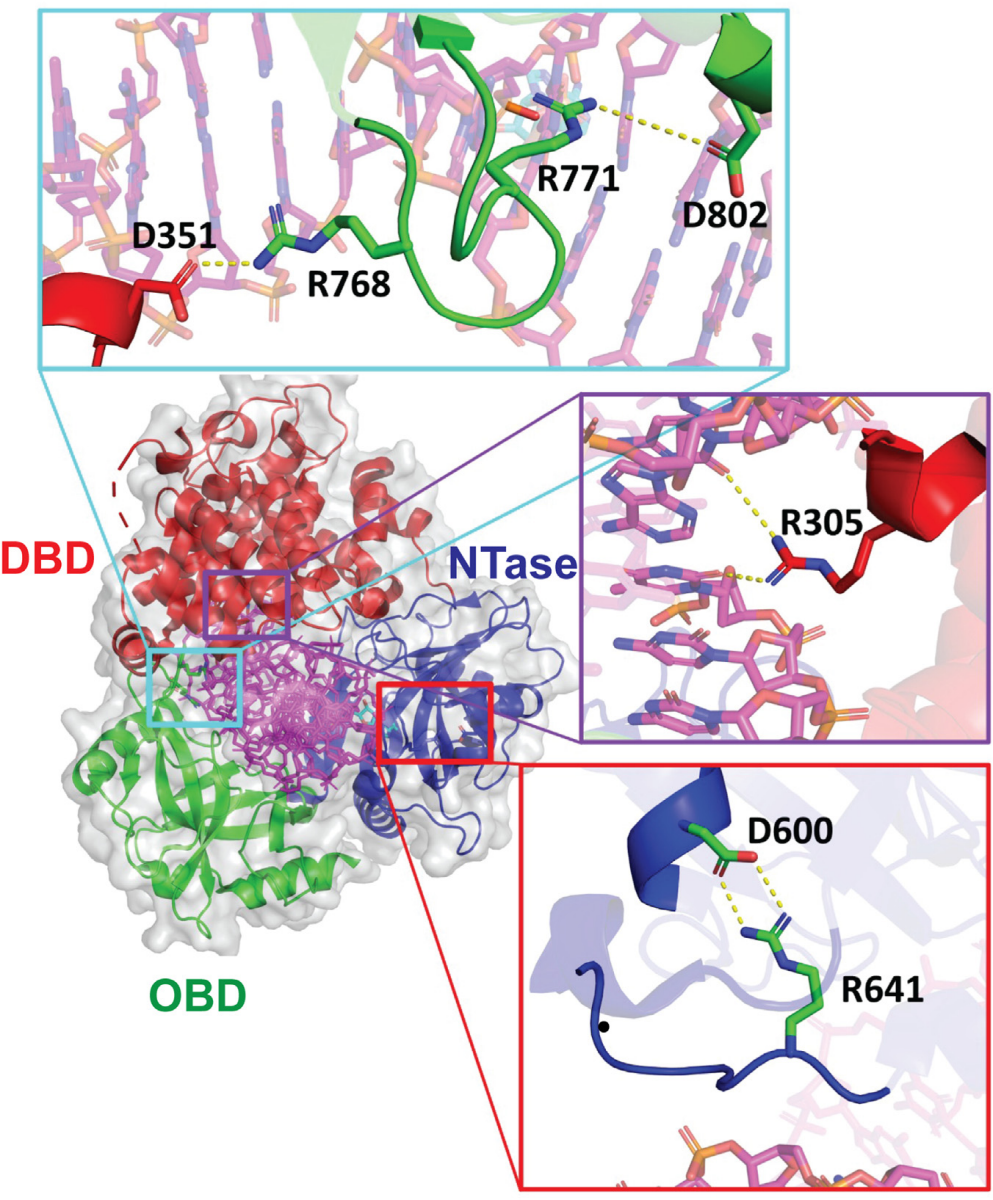
**LIG1 structure highlighting residues of interest.** The crystal structure of Δ262 LIG1 bound to nicked DNA (PDB 6P0C; (14)) consists of the DBD (*red*), the NTase domain (*blue*), and the OBD (*green*). The insets highlight residues of interest. The OBD contains a DNA binding loop with two arginine residues at positions 768 and 771. R771 interacts with the DNA, and it also makes a salt-bridge interaction with D802 within the OBD. R771 makes a salt-bridge interaction across the interface to the DBD. Within the DBD, R305 makes a network of interactions with the minor groove of the DNA. R641 forms a salt-bridge interaction with D600 within the NTase domain.

It was expected that the R641S variant would have a biochemical phenotype similar to the previously characterized R641L variant. Indeed, they both decrease the activity of LIG1 and increase the K_M, DNA_ value when compared to the WT enzyme. The effects of both variants are partially dependent on the Mg^2+^ concentration since not only is the affinity towards Mg^2+^ weakened but increasing Mg^2+^ concentration to saturating conditions almost completely rescues the defect in K_M, DNA_. However, the biochemical defects of R641S LIG1 are more severe than they are for the R641L variant at the physiological Mg^2+^ condition. The catalytic efficiency of R641S is ∼6-fold lower than R641L (Fig. 7*A*). Additionally, R641S shows elevated abortive ligation at physiological Mg^2+^ concentration of 1 mM, and this persists at the elevated Mg^2+^ concentration of 20 mM, a condition that rescues the abortive ligation for the R641L variant (Fig. 5*A*). Consideration of the previously published structure of R641L LIG1 in complex with DNA provides a rationale for the greater deleterious effects of R641S (19). The leucine substitution disrupts a salt-bridge interaction with D600 (Fig. 8) leading to a complete rearrangement of the DNA binding loop and moving it further from the phosphodiester backbone. The leucine is accommodated by a newly formed hydrophobic pocket. Such an accommodation is unlikely for the polar serine side chain, presumably leading to a unique rearrangement. A structure of the R641S variant would be needed to address the details of how this loop rearranges; however, it is likely that the closed conformation of LIG1 is further destabilized by these rearrangements relative to the rearrangements in R641L. Interestingly, R641C has also been reported as a rare LIG1 variant (Table S2), although we did not investigate this allele in the current work. Given the strong defects with both the R641S and R641L substitutions, we predict that the R641C allele will also be strongly deleterious.

### R768W is less defective than R771W

Both R768 and R771 are located on a DNA binding loop of the OBD which intercalates into the minor groove of the DNA helix. R771 makes a salt-bridge with D802 while R768 makes a salt-bridge with D351 in the DBD. The latter salt bridge helps stabilize the interdomain interactions between the OBD and the DBD required for ring closure around the DNA substrate (Fig. 8). The structure of the R771W variant leads to local conformational changes that cause the loss of both salt bridges (16). We hypothesized that the R768W substitution would disrupt the salt bridge with D351 and lead to rearrangements that are analogous to those found in the R771W variant. However, the biochemical consequences of the R768W substitution differ from the R771W substitution. Despite similar defects in catalytic efficiency due to decreased k_cat_ values and increased K_M, DNA_ values, and the similar partial rescue by super-physiological, saturating Mg^2+^ concentrations (Fig. 7), R768W does not exhibit the severe abortive ligation that is seen with the R771W variant (Fig. 5*A*). This suggests that unique conformations in the interdomain interactions of the OBD and the DBD alter the propensity to dissociate from the AMP-DNA intermediate. The R771W variant is likely more disruptive of the closed ring structure of LIG1, which could account for the increased propensity to release the AMP-DNA intermediate as compared to the R768W variant. Regardless of the unique molecular rearrangements in the R768W and R771W variants, the difference in abortive ligation will provide a unique opportunity to evaluate the impact of abortive ligation in a cellular context.

### R305Q uniquely impacts DNA binding affinity

The final variant, R305Q, is in the DBD where it makes a network of interactions with the minor groove of the DNA (Fig. 8). The arginine to glutamine substitution results in the loss of charge and is expected to weaken the interactions with DNA. Biochemical characterization shows only moderate defects in the maximal rate constant for DNA ligation (k_cat_) at physiologically relevant concentrations of Mg^2+^ and at saturating concentration of Mg^2+^ this defect is fully rescued (Table 2). However, the K_M, DNA_ value at both physiological and saturating Mg^2+^ concentrations is between 8- and 16-fold higher for R305Q than for the WT enzyme (Table 2). Although the other LIG1 Syndrome variants have decreased K_M,__DNA_ values at saturating Mg^2+^, the value for R305Q is insensitive to the concentration of Mg^2+^ and remains high at saturating concentration of Mg^2+^ (Table 2). In the absence of Mg^2+^, we were able to directly measure DNA substrate binding by each of the LIG1 variants (Fig. 6). With this assay, R305Q has an approximately 40-fold defect in binding to nicked DNA which is in contrast with the other tested mutations that do not substantially weaken DNA binding. Despite the clear defect in DNA binding, the R305Q mutant does not exhibit abortive ligation. This is unexpected because abortive ligation involves the dissociation of LIG1 from the AMP-DNA intermediate. It appears that the DBD makes different contributions to DNA binding in the different steps of the ligation reaction, and it is interesting to consider that it may be the disengagement of the NTase domain/OBD from the AMP-DNA intermediate that controls abortive ligation. However, it will be important to investigate additional substitutions in the DBD to evaluate if this observation is indeed generalizable or whether it is a unique property of the R305Q substitution.

### Implications for LIG1 syndrome

While each of the mutants we tested here exhibited different types and levels of defectiveness, we have found that all three are likely candidates to cause LIG1 Syndrome if they were to be present in a biallelic context. At physiological concentration of free Mg^2+^ (1 mM), the R305Q and R768W substitutions have large deleterious effects on the catalytic efficiency for DNA ligation that are comparable to the previously described R641L and R771W variants (Fig. 7). In contrast, R641S has stronger deleterious effects than the previously described LIG1 Syndrome variant R641L. The more severe biochemical defects associated with R641S as compared to the other LIG1 Syndrome mutations raise the possibility that this variant could be partially dominant. Although it would have difficulty competing against the WT enzyme, given the substantial decrease in k_cat_/K_M_ for the R641S variant, it invariably results in abortive ligation under physiological conditions which would serve as a strong block to the activity of the WT enzyme. However, this is speculation, and we are not aware of whether any immune-compromised patients do not conform to a biallelic presentation (5, 20, 23, 24).

Each of these new LIG1 variants is rescued by increasing the concentration of free Mg^2+^ to a saturating concentration of 20 mM (Fig. 7). This is the same pattern that was observed for the R641L and R771W variants and a corollary of this Mg^2+^ effect is that all these putative LIG1 Syndrome variants are predicted to be very sensitive to the concentration of free Mg^2+^ in the physiological range (5). Although there are no pharmacological means to elevate intracellular Mg^2+^ levels above-normal levels, Mg^2+^ deficiency can readily be treated by supplementation and therefore it would be beneficial to monitor patient Mg^2+^ levels to avoid dietary magnesium deficiency which is expected to worsen the impact of the LIG1 variant alleles.

The unique biochemical characteristics of R305Q and R768W have new implications for understanding LIG1 Syndrome. Until now, the defective mutants believed to lead to LIG1 Syndrome shared similar biochemical characteristics. The increase in abortive ligation for R641L and R771W was thought to be a contributing factor to the emergence of LIG1 Syndrome symptoms. However, there is now an example of a homozygous mutation (R305Q) that is clearly causing LIG1 Syndrome-like symptoms (20) without causing an increase in abortive ligation. One of the defining characteristics of R305Q is a very large defect in DNA binding which was not seen in the other variants. Thus, there is more than one mechanism leading to LIG1 Syndrome, and the accumulation of the abortive intermediate may not be clinically significant. Although R768W has not been validated as a *bona fide* LIG1 syndrome variant, it too shares the property with the R305Q variant of decreased catalytic deficiency without elevated abortive ligation. In the case of the R305Q variant, the large defect in DNA binding could uniquely alter the workload between LIG1 and other DNA ligases in the cell and change the fidelity of DNA replication and repair. The most direct way to investigate this hypothesis will be future studies of animals carrying the R305Q allele to evaluate if there are additional impacts beyond the immune system, such as the predisposition to cancer that was previously described in the R771W variant mice (25).

It took almost 30 years from the time that the initially reported LIG1 Syndrome patient was described in 1992 (6, 7) before additional LIG1 Syndrome patients were reported (5). With the widespread prevalence of whole exome sequencing, it is expected that the rate of discovery of new LIG1 syndrome alleles will be accelerated from the cohort of patients suffering from immune deficiency. Indeed, there have been recent publications describing two additional patients with novel LIG1 disease variants (23, 24).^1^ Given the central role of LIG1 in DNA replication and repair, it is somewhat surprising that catalytically compromised mutations in LIG1 are tolerated through development and that the predominant phenotype of LIG1 deficiency lies in the differentiation of immune cells. The mechanism by which LIG1 deficiency impacts the immune system can be addressed by both clinical characterization of additional patients and by the quantitative comparison of the biochemical properties of these alleles. The unique biochemical properties of the R305Q allele as compared to the R771W and R641L alleles will be particularly informative in evaluating the consequences of LIG1 mutation and eventually lead to better predictors of how unannotated LIG1 variants may impact clinical phenotype.

## Experimental procedures

### Preparation of DNA

Oligonucleotide substrates were purchased from Integrated DNA Technologies or from the Keck Oligo Synthesis Resource at Yale University, purified by denaturing polyacrylamide gel electrophoresis (PAGE), and the concentration determined from the theoretical extinction coefficient at 260 nm as previously described (15). DNA sequences are provided in Table S1. To anneal nicked DNA substrates, three strands were mixed at a ratio of 3:2:1 (template: 3′-OH-strand: 5′-phosphate-strand) in an annealing buffer with 10 mM NaMES pH 6.5 and 50 mM NaCl. The solution was heated to 95 °C in a thermocycler and cooled to 4 °C at a rate of 3 °C per minute.

### Preparation of LIG1 protein

The catalytic domain of human LIG1 (232–919) was expressed from a pET19-based plasmid (8). Each Δ232 LIG1 mutant vector was made using site-directed mutagenesis and confirmed by sequencing both strands of the coding region. LIG1 WT and variants were expressed and purified according to the previously described protocol (5). Purified LIG1 proteins were dialyzed in 50 mM Tris-HCl, pH 7.5, 150 mM NaCl, 1 mM DTT, and 0.1 mM EDTA and stored at −80 °C. The absorbance at 280 nm was used to estimate the protein concentrations of each protein. The extinction coefficients used for the calculation of the LIG1 concentrations were 65,320 M^−1^ cm^−1^ for the R768W variant and 59,820 M^−1^ cm^−1^ for the other variants and WT LIG1. LIG1 is fully adenylylated when purified according to this protocol (5). Active concentrations of the enzymes were determined *via* active site titration in the absence of ATP whereby increasing concentrations of LIG1 are incubated with 150 nM fluorescein-labeled 28mer nicked DNA substrate. At least two independent purifications were performed for each variant and the steady-state ligase activity was not significantly different between preparations. As UV-absorbing impurities can lead to an overestimate of protein concentration, we used the active site titrations to calculate the respective enzyme concentrations throughout this manuscript.

### Gel-based ligation assays

The ligation assays were performed at 37 °C in a standard reaction buffer containing 50 mM NaMOPS at pH 7.5, 0.05 mg/ml BSA, 1 mM dithiothreitol, and enough NaCl to maintain an ionic strength of 150 mM. The concentrations of ATP, MgCl_2_, the 28-mer substrate, and LIG1 depended on the experiment as indicated below. The free magnesium concentration was calculated based on the dissociation constants for the Mg^2+^•ATP complex for any reaction containing ATP (15). The reactions were quenched with standard loading buffer (90% formamide/50 mM EDTA/0.01% bromophenol blue/0.01% xylene cyanol). After quenching, samples were heated to 95 °C and loaded onto a 15% (w/v) polyacrylamide gel containing 8 M urea to separate the fluorescein-labeled substrate and product. These were detected using an Amersham Typhoon five imager (Cytiva), and the gel images were analyzed with ImageQuant TL software (GE). The fraction of ligated product was determined for each time point according to Equation 1 where F_p_ is the fraction product, and p, s, and i are the intensities of the bands for product, substrate, and AMP-DNA intermediate.

(1) $$F_{p}=\frac{p}{p+s+i}$$

### Preincubation controls to determine the stability of LIG1 variants

To test if LIG1 variants were sufficiently stable for kinetic analysis, we incubated each of the purified proteins alongside WT LIG1 for periods up to 2 h. At various time points, DNA substrate was added, and steady-state ligation assays were used to determine the initial rates. To account for the differences in steady-state ligation activity the amount of enzyme was 2 (WT and R768W), 5 (R305Q), or 43 (R641S) nM enzyme. The incubation conditions were the standard reaction conditions with 0.2 mM ATP and 1.2 mM MgCl_2_ (1 mM Mg^2+^_free_). After the indicated incubation time, reactions were initiated by the addition of 1000 nM DNA substrate.

### Characterization of steady-state kinetics

Reactions for steady-state dependencies were carried out in a standard reaction buffer with 0.5 to 50 nM LIG1. To measure the dependence on Mg^2+^ and ATP concentration, we used a 1000 nM 28-mer DNA substrate. To measure the ATP dependence the reactions included 20 mM free Mg^2+^ and 1 to 200 μM ATP. For determination of the Mg^2+^ dependence, the reactions contained 200 μM ATP and 0.25 to 20 mM free Mg^2+^. Finally, the DNA dependence was determined with 10 to 2000 nM 28-mer DNA substrate and at two different conditions: physiological, 1 mM free Mg^2+^ (1.2 mM MgCl_2_ and 200 μM ATP) or saturating (20 mM free Mg^2+^ and 1 mM ATP) conditions. All steady-state reactions had their initial rates determined *via* linear regression which were then fit by the Michaelis-Menten equation (Equation 2):

(2) $$\frac{V_{init}}{\left[E\right]}=\frac{V_{\max}\left[S\right]}{K_{M}+\left[S\right]}$$

Initial rates for reactions in which both intermediate and product accumulated were analyzed based on the total disappearance of the substrate. Therefore, they reflect the kinetics for substrate binding and adenylyl transfer regardless of whether ligation is efficient or results in abortive ligation. We determined the fraction of abortive ligation events by taking the rate of intermediate accumulation (V_intermediate_) and dividing it by the rate of total substrate disappearance (V_product_ + V_intermediate_) as in Equation 3:

(3) $$Fraction\;abortive\;ligation=\frac{\left(V_{intermediate}\right)}{\left(V_{Product}+V_{intermediate}\right)}$$

### Steady state DNA binding assays

Equilibrium binding of LIG1 to a nicked DNA substrate was performed in the absence of Mg^2+^ to prevent ligation (19). Remarkably many ligases tolerate a 3′-fluorescein label when placed off the five-position of T (TFAM) (19, 26). Measurements of fluorescence intensity were collected with a FluoroMax 3 fluorometer (Horiba) with DataMax software. Data was collected with an excitation wavelength of 495 nm and an emission wavelength of 515 nm with a bandpass of 6 nm. Binding assays were performed at 37 °C by titrating in increasing amounts of LIG1 to a 2.5 ml solution with 4 nM of the TFAM-44mer. The standard reaction buffer with the addition of 0.2 mM EDTA was used. Reactions were allowed to equilibrate for 30 s to 1 min after LIG1 was added before the measurements were taken. The relative fluorescence is plotted as a function of the concentration of LIG1 and fit by a hyperbolic binding curve shown in Equation 4 to determine the LIG1 binding affinity (K_D_) for the DNA substrate (y is the relative fluorescence intensity, Y_free_ is the fluorescence intensity of the free DNA and ΔY is the change in fluorescence upon binding of LIG1).

(4) $$y=Y_{free}-\frac{\Delta Y\left[LIG1\right]}{K_{D}+\left[LIG1\right]}$$

## Data availability

All data can be found within the manuscript and accompanying supporting information. This study produced several expression plasmids that are available upon request.

## Supporting information

This article contains supporting information (27).

## Conflict of interest

The authors declare that they have no conflicts of interest with the contents of this article.

## Footnotes

1 While this manuscript was under review, another study was published regarding the R305Q variant. Their findings independently confirm the weakened DNA affinity and reduced catalytic activity of this variant that is described herein (https://doi.org/10.1016/j.jmb.2024.168813).
