# Supplementary material for: Rare variants of DNA ligase 1 show distinct mechanisms of deficiency
Source: J Biol Chem. 2024 Nov 5;300(12):107957. doi: 10.1016/j.jbc.2024.107957 (PMC11648212; doi:10.1016/j.jbc.2024.107957)
Supplement: Supplemental Tables S1 and S2, and Fig. S1 [file mmc1.pdf]

Supporting Information

Rare Variants of DNA Ligase 1 Show Distinct Mechanisms of Deficiency

Jenna H. Veenstra, Alexandria Chabez, Terrance J. Haanen III, Austin Keranen, Charlotte Cunningham-Rundles, and Patrick J. O'Brien

Correspondence: pjobrien@umich.edu

Table of Contents

Table S1. Oligonucleotides for ligation and binding assays p. S2
Table S2. LIG1 allele frequency p. S2
Figure S1. Steady-state ATP dependence for LIG1 variants p. S3

Table S1. Oligonucleotides for ligation and binding assays

Oligo Name	5' Mod	Sequence	3' Mod
Up13OH		5' GTGCTGATGCGTC 3'	
DownP15-FAM	Phosphate	5' P-GTCGGACTGATTCGG-FAM 3'	Fluorescein (6-FAM)
Temp28		5' CCGAATCAGTCCGACGACGCATCAGCAC 3'	
Up22-TFAM		5' CGCCGCGCATTATAATATTAATFAM 3'	Fluorescein-dT (TFAM)
Down22	Phosphate	5' P-AATATAAATATAATCGCCGCGA 3'	
Temp44		5' TCGCGGCGATTATATTTATATTATTAATATTATAATGCGCGGCG 3'	

Table S2. LIG1 Allele Frequency

Variant	MAF[a]	Reference[b]
R768W	3.9e-5	(18)
R771W	2.6e-5	(5)
R641L	5.8e-5	
R641S	Unknown	ND
R641C	1.3e-5	(18)
R305Q	2.5e-6	(16)

[a]Minor allele frequency (MAF) from the gnomAD database v4.1.0 (21). [b]Reference describing clinical diagnosis of patients harboring this allele. ND, not described in the literature.

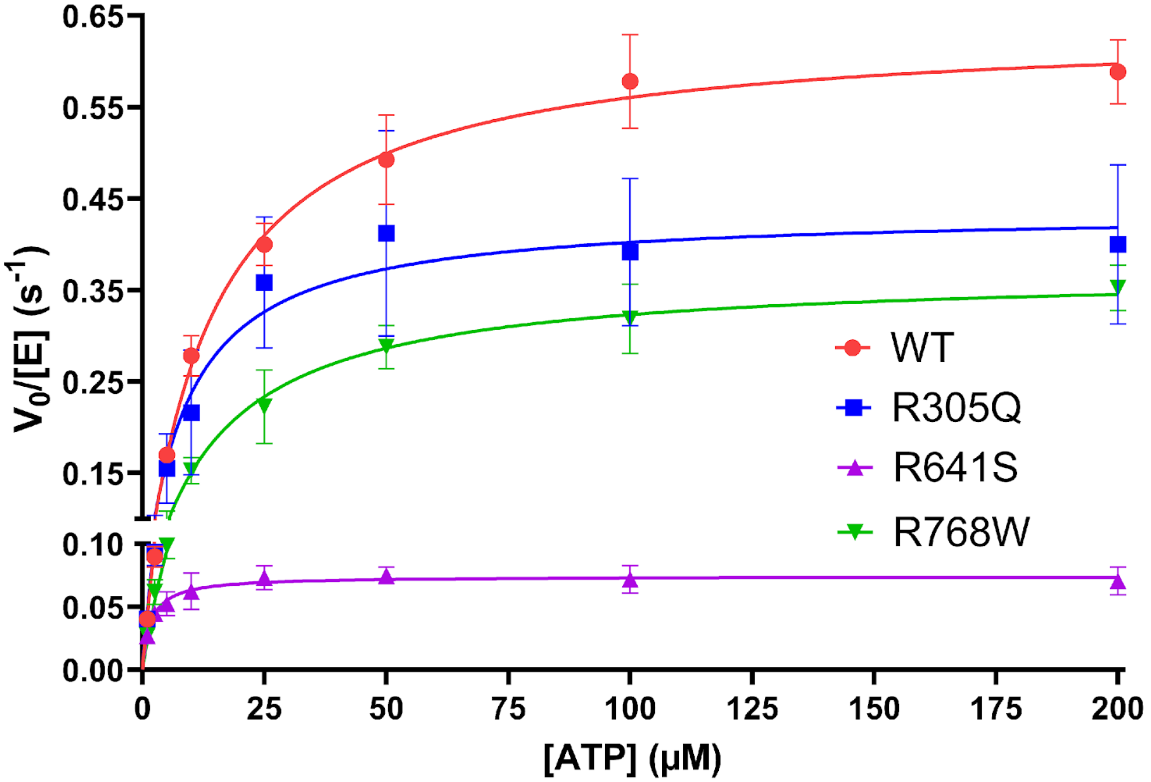

Figure S1. Steady-state ATP dependence of LIG1 variants. Initial rates were determined with 1 – 10 nM LIG1, 1000 nM DNA substrate, 20 mM free Mg^{2+}, and increasing amounts of ATP at 37 °C. These data were fit by the Michaelis-Menten equation to obtain k_{cat} and $K_{M,ATP}$ values for WT and variant LIG1 enzymes. Values are the mean ± SD (N ≥ 3) and the kinetic parameters are presented in **Table 1**.
